# First successful transcatheter valve-in-valve implantation into a failed mechanical prosthetic mitral valve after fracturing the discs: a case report

**DOI:** 10.1093/ehjcr/ytaf183

**Published:** 2025-05-09

**Authors:** Christian Butter, Michael Neuss, Tanja Kücken, Doreen Bensch, Michael Erb

**Affiliations:** Department of Cardiology, Heart Centre Brandenburg Bernau and Faculty of Health Sciences Brandenburg, Brandenburg Medical School (MHB) Theodor Fontane, 16321 Bernau/Berlin, Ladeburger Straße 17, Germany; Department of Cardiology, Heart Centre Brandenburg Bernau and Faculty of Health Sciences Brandenburg, Brandenburg Medical School (MHB) Theodor Fontane, 16321 Bernau/Berlin, Ladeburger Straße 17, Germany; Department of Cardiology, Heart Centre Brandenburg Bernau and Faculty of Health Sciences Brandenburg, Brandenburg Medical School (MHB) Theodor Fontane, 16321 Bernau/Berlin, Ladeburger Straße 17, Germany; Department of Radiology, Heart Centre Brandenburg Bernau, 16321 Bernau/Berlin, Ladeburger Straße 17, Germany; Department of Cardiothoracic Surgery, Heart Centre Brandenburg Bernau, Brandenburg Medical School (MHB) Theodor Fontane, 16321 Bernau/Berlin, Ladeburger Straße 17, Germany

**Keywords:** Case report, Transcatheter mitral valve-in-valve implantation, Degenerated mechanical valve, Valve fracture, Cerebral protection, Carbon fragment embolisation

## Abstract

**Background:**

Until now, dysfunctional mechanical valves had to be treated surgically. Motivated by *in vitro* fracture experiments and the first successful clinical implantation of a transcatheter valve (TAVR) into the remaining ring in aortic position, this approach was considered for the mitral position for the first time.

**Case summary:**

A 31-year-old female patient with a history of four open-heart surgeries and severe neurologic complications presented with cardiac decompensation due to a fixed tilt of her mechanical bileaflet mitral valve prosthesis, resulting in mitral stenosis with a mean gradient of above 10 mmHg. An interventional approach was discussed. Using an apical access, the tilts were cracked under cerebral protection, and a balloon-implantable TAVR was implanted uneventfully. More than 3 years later, the patient is clinically stable, the valvular function is not impaired and the embolized fragment does not cause any problems in the distal abdominal aorta.

**Discussion:**

To the best of our knowledge, this is the first case report that demonstrates the possibility to implant a biological TAVR in a failed bileaflet mechanical mitral valve after fracturing the carbon tilts.

**Learning points**

- Until now, only open surgical replacement was possible in non-functioning mechanical heart valves.
- We demonstrate that interventional transcatheter replacement can be performed on a mechanical mitral valve after protected fracturing.
- This new approach might offer a treatment option for a small, selected group of patients for whom surgical treatment is not an option.

## Introduction

Transcatheter mitral valve replacement has been widely investigated in different settings (valve-in-valve, valve-in-ring and valve-in-MAC). However, replacement had been never performed with a mechanical valve.^1^ Treatment of a mechanical valve is limited to surgical replacement, which is a redo procedure and is associated with doubling of mortality (4.7% vs. 2.2%).^2^ The proportion of patients who develop long-term valve dysfunction of a mechanical valve is around 2%.^3^ Of these patients with acute valve dysfunction, around one third die before or during a possible re-intervention.^3^ Motivated by our initial experience^4^ with successful implantation of a transcatheter balloon-expandable valve into a dysfunctional and intentionally fractured aortic bileaflet valve (SJM® AHPJ prothesis 23 mm) using a high-pressure balloon (TRUE Dilatation 20 mm—RBP 16atm; LOMA Vista) and with the patient’s written informed consent for publication, we chose the same approach for the first time in a mechanical mitral valve in a patient deemed surgically untreatable by independent heart teams.

In this case report, we present clinical and procedural aspects as well as 3-year follow-up data of the first intentional fracture of a dysfunctional mechanical valve in the mitral position in humans.

## Summary figure

**Figure:**
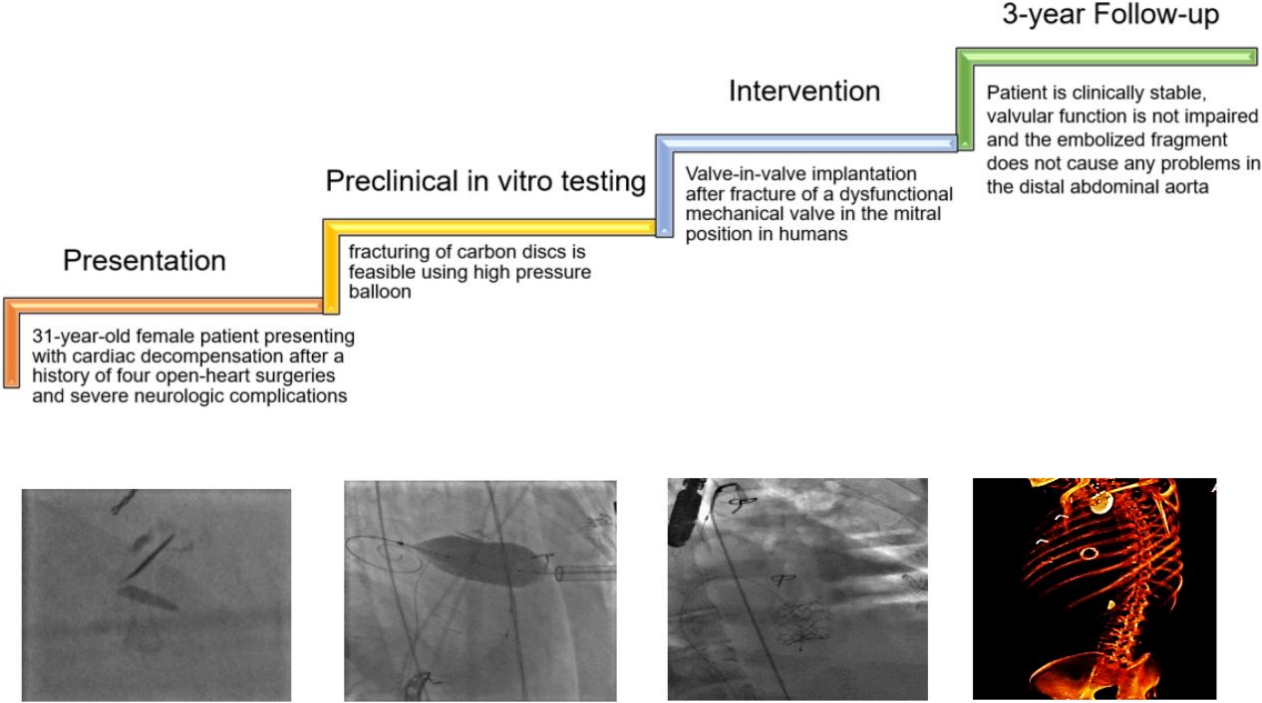


## Case summary

A 31-year-old Caucasian female presented with severe dyspnoea, signs of congestion on chest X-ray and weight gain of 4 kg in the last few days. NYHA class was III-IV, BMI 29.6. The physical and cardiovascular examination showed coarse crackles, irregular heartbeat and bilateral lower leg oedema. On admission, blood pressure was 90/65 mmHg. Oxygen saturation was 88% without oxygen. The suspected high-frequency clicking above the mitral valve was not audible.

Her dyspnoea had worsened over the past few weeks and she was not responding to outpatient treatment for heart failure. She had a long history of complex and critical cardiac surgery with severe neurological complications (see Supplementary material online, *Suppl. S1*).

**Figure 1:**
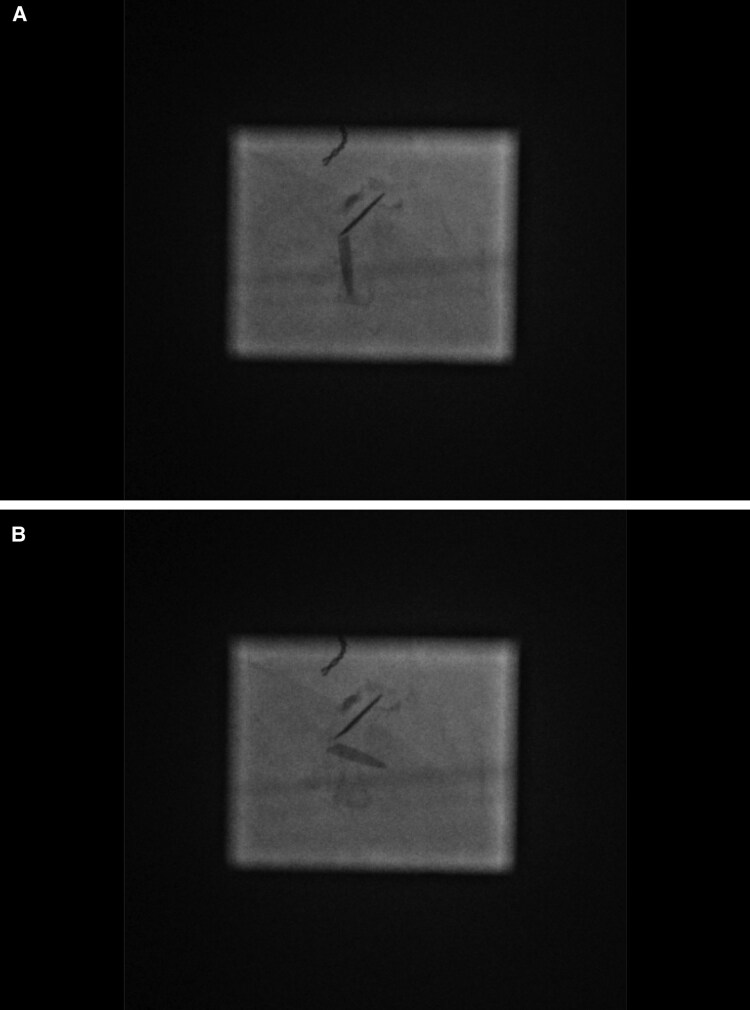
Unchanged fixed position of the upper disc during systole (*A*) and diastole (*B*) of the bileaflet mechanical mitral valve.

**Figure 2:**
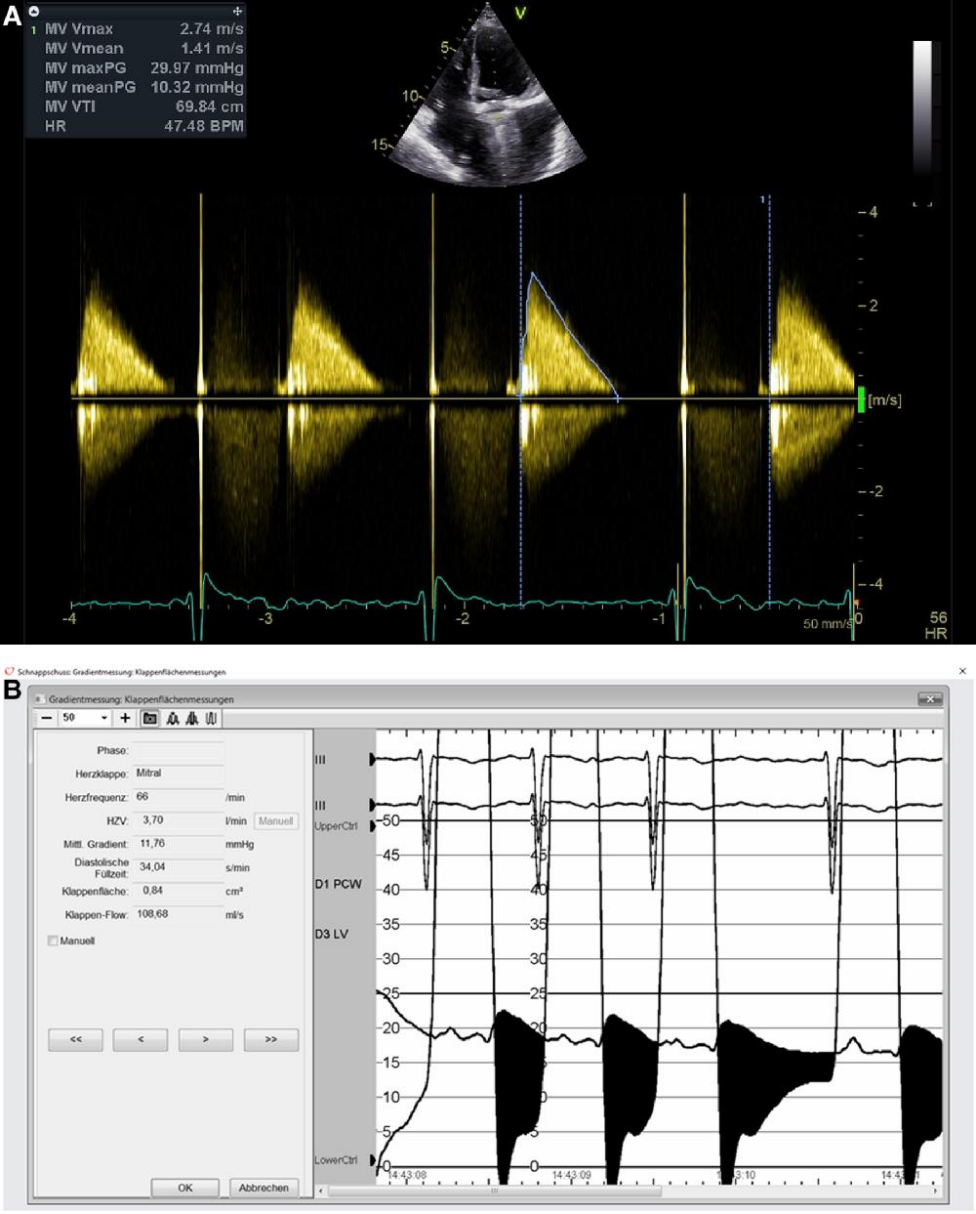
Doppler gradient of 10.32 mmHG above mitral prothesis (*A*); confirmed in invasive parallel pressure measurement (*B*).

A malfunction of her mechanical bileaflet mitral valve (St. Jude Medical 29 mm implanted 1996) with fixation of a disc, demonstrated under fluoroscopy and echo (*Figures 1* and *2*), was considered to be the cause of severe mitral stenosis with a Doppler gradient of 11mmHG and a pressure half time based opening area of 1.6 cm^2^. Pulmonary pressure (34/16/22 mmHg), left ventricular ejection fraction, as well as RV function (TAPSE 15 mm) were near normal. The left atrium was moderately enlarged. The electrocardiogram showed a well-regulated permanent atrial fibrillation with a ventricular rate of 69/min and a normal QRS width. NT-proBNP on admission was 2046 pg/dL (normal value <190 pg/dL). There were no other abnormal laboratory parameters.

The patient was presented to two independent cardiac surgery centres, which estimated the perioperative mortality as very high (EuroScore II 17.99%, Society-of-Thoracic-Surgeons Score 13.68%). Both rejected the patient. Planning for the procedure included a careful review of the existing operation protocol describing the difficulties and anatomical challenges, computed tomography (CT) scan of potential problems in surgical access and personal discussions with the patient and her family. The heart teams consisted of a cardiac surgeon, an anaesthetist experienced in complex cardiac surgery, a paediatric cardiac surgeon and a team of interventional cardiologists. An apical access was chosen, and a temporary pacemaker was installed. Cerebral protection was achieved using the SENTINEL (Boston Scientific) device via right brachial artery. The mechanical valve was easily passed in the centre with a Terumo wire steered by a multipurpose diagnostic catheter, and the wire was replaced with a Safari S wire (Boston Scientific). An initial attempt to place a True^TM^ Dilatation 24 × 45 mm balloon (Bard) failed due to its profile and instead an Atlas^TM^ Gold 24 × 40 mm (Bard) was successfully placed. Under rapid pacing only 5 atm were required for the successful fracture of both carbon discs. The prepared Sapien 3–26 mm (Edwards) valve was placed under TEE guidance and deployed under rapid pacing. Immediate full function with a tiny paravalvular leak was confirmed by TEE. Surprisingly, the complete loss of any valvular function did not result in any haemodynamic instability, it was well tolerated, and no inotropic support was needed. Only tiny pieces of white tissue, but no carbon fragments, were found in the cerebral protection filters. A critical review of high-resolution intraprocedural fluoroscopy revealed a shadowy ‘butterfly-like’ structure moving for a few beats in the left atrium.

**Figure 3:**
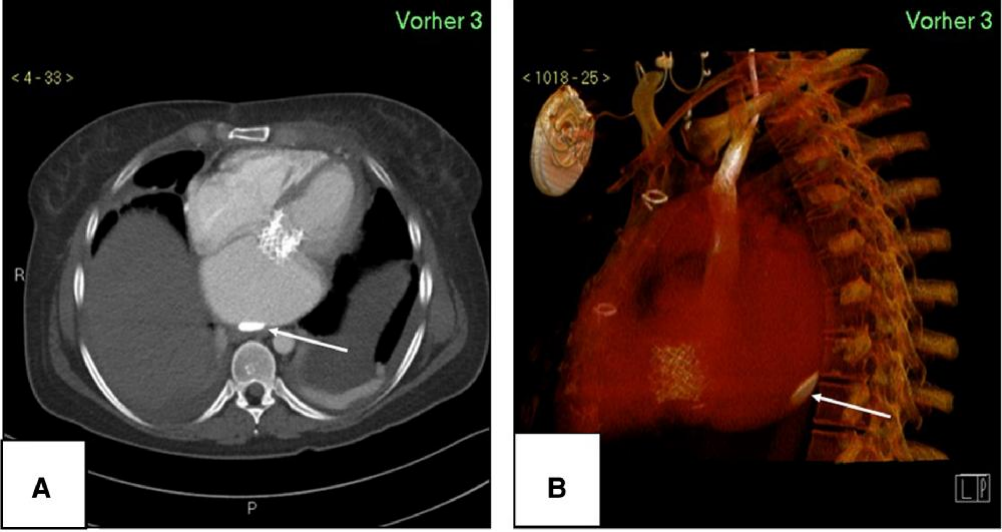
Chest CT taken in supine position in transverse plane (*A*) and sagittal plane (*B*) showing the tilt (white arrow) in the inferior-posterior left atrium.

Angiograms of the aortic root, cerebral arteries, abdominal aorta, mesenteric arteries and the iliac and femoral arteries were performed to localize the fractured discs and to exclude perfusion abnormalities. Neither a fragment nor a perfusion abnormality was detected. TEE on day one after the procedure showed a hyperdense structure adherent to the wall of the left atrium. A low-dose chest CT in supine position was performed, identifying the structure as a fully preserved disc projected onto the spine (*Figure 3*).

**Figure 4:**
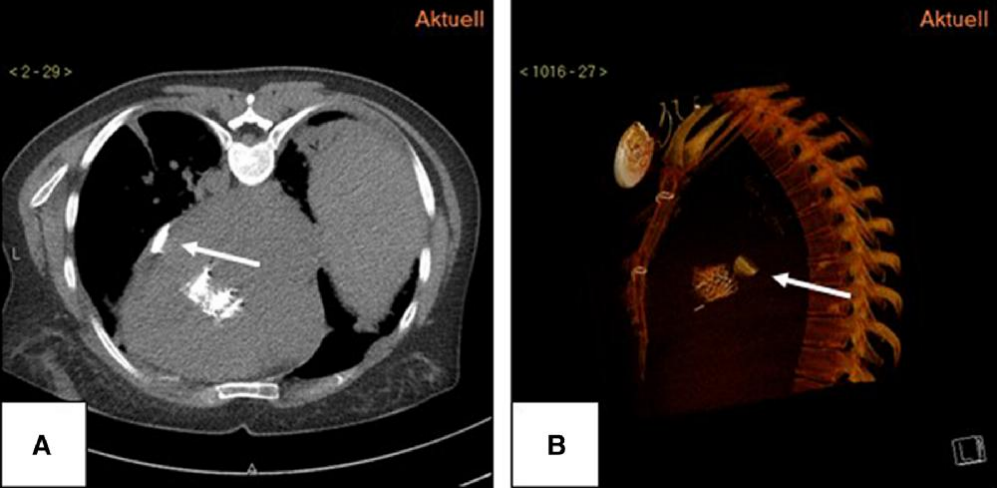
Chest CT taken in prone position in transverse plane (*A*) and sagittal plane (*B*) showing the tilt (white arrow) in the antero-lateral left atrium.

**Figure 5:**
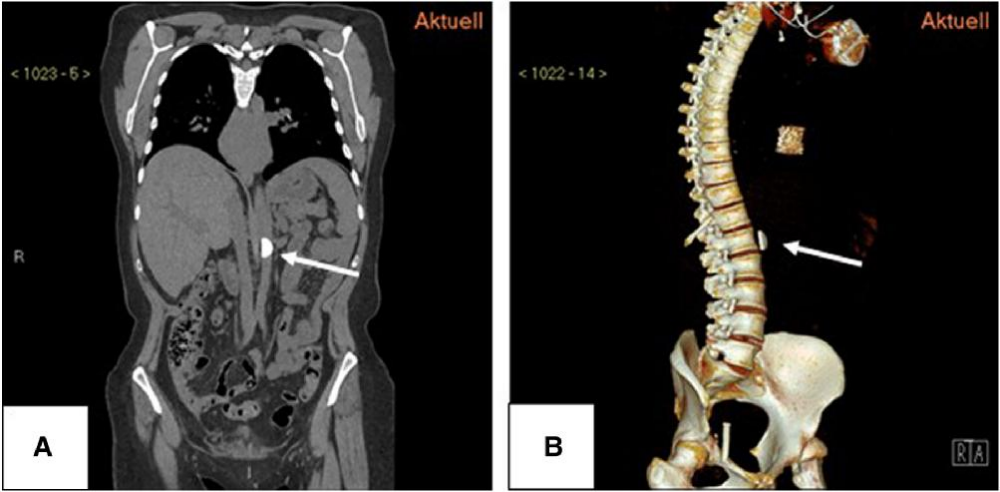
CT scan of the chest and abdomen in frontal plane (*A*) and oblique (*B*) showing the tilt (white arrow) in abdominal aorta.

The CT scan was repeated a few days later in the prone position to exclude possible embolisation (*Figure 4*). The disc had moved according to gravity and was located on the anterior lateral side of the left atrium. Both images in the prone position were flipped to facilitate anatomical comparison with the supine position. A further control CT a few months later localized the disc in the abdominal aorta above the renal arteries in the direction of flow (*Figure 5*). Perfusion below the disc was not impaired. The second disc was never localized. The clinical situation is still stable after 3 years, and her activity, which is limited by her severe neurological disability, has further improved. The mean pressure gradients over the biological mitral prosthesis were 7.57 mmHg at 12 months, 8.22 mmHg at 24 months, and 7.54 mmHg at 36 months. NT-proBNP was stable over time with values between 753 and 1524 ng/dL. No clinical complaints were reported, and no gastrointestinal or renal dysfunction occurred. Ultrasound and Doppler examinations of the abdominal aorta and descending arteries showed no flow restrictions. Protected by oral anticoagulation, no embolic events or bleedings occurred. Further images and a video summarising the intervention are shown in Supplementary material online, *Suppl. S2*.

## Discussion

Mechanical heart valves have a long surgical history in the replacement of dysfunctional valves, especially in the aortic and mitral positions in younger patients, due to their mechanical longevity and missing degeneration. The first mechanical bileaflet St. Jude valve was implanted in a human patient in 1977. Since then, more than 1.3 million valves have been implanted worldwide, in 2023 ∼2000 in Germany.^5^ In case of a rare malfunction, surgical replacement was the only option.

On the other hand, biological transcatheter valve (TAVR)-in-valve implantations in degenerated bioprothesis have been established. Motivated by an *in vitro* simulation and our first successful clinical experience fracturing a malfunctioning bileaflet prosthesis in aortic position in transcatheter technique with a high-pressure balloon and directly implanting a biological TAVR,^4^ we chose the same approach to replace a malfunctioning mitral valve.

Here, we describe a 31-year-old female with a complex history of multiple open-heart surgeries presenting with cardiac decompensation. A transapical access was used to reach the mitral valve over a short distance, and because we were concerned about a difficult transseptal puncture due to three previous operations involving the interatrial septum, the extensive scar tissue described, the small body size and the potential need for rapid valve-in-valve implantation after a disc fracture. We used the same cerebral protection device as in our initial aortic case,^4^ although one could debate whether this is the best tool. The fully open mitral valve did not cause any haemodynamic instability after fracture. Compared with our previous experience with the aortic valve and the very low ejection fraction, the near-normal left and right ventricles might be responsible for the better haemodynamic tolerance.

The tilts were cracked under cerebral protection, and a balloon-implantable TAVR was implanted uneventfully. In the present case, we suppose that the fixed and finally fractured immobile tilt remained adherent to the ring, the movable part was fractured in both peripheral hinges, but the rest of the body was completely undestroyed. No tiny fragments were found in the cerebral protection filters. Thus, the disc passed clinically unnoticed through the valve-in-valve and the native aortic valve and embolized into the abdominal aorta. The major concern of fracturing a mechanical valve is the risk of embolisation and the final location of the fragments, which might cause either an embolic stroke or peripheral malperfusion. CT scans demonstrated that the tilt remained in the left atrium, initially posture-dependent, but subsequently passed undetected through the new biological mitral valve as well as the aortic valve until it was fixed in the abdominal aorta at the level of the renal artery for more than 3 years. The location and possible removal of the disc would have required major abdominal surgery, which the patient denied.

The second disc was never localized. We suppose that it was fixed in the scar tissue between the mechanical ring and the Sapien 3.

## Conclusion

In conclusion, in the rare case of non-functioning mechanical heart valves and after careful discussion and refusal of cardiac surgery options, TAVR-in-valve implantation may be an option after tilt fracture, even in the mitral position. Embolisation of the fragments is still the riskiest and most unpredictable part. Even after acute protection of the brain, delayed, clinically unnoticed embolisation into the abdominal aorta was discovered by chance on CT. Early CT scan after the acute intervention should be encouraged and repeated until a final and fixed destination has been reached.

## Lead author biography


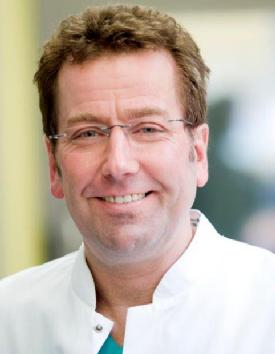


Prof. Christian Butter is Head of the Department of Cardiology at the Heart Center Brandenburg in Bernau/Berlin (Germany). In 2015, he was appointed professor for cardiology at the Brandenburg Medical School. At the beginning of his clinical and scientific carrier he focused on device therapy and heart failure. Today, he is deeply involved in the development and interventional therapy of structural and valvular heart disease.

## Supplementary Material

ytaf183_Supplementary_Data

## Data Availability

All data generated or analysed are included in this published article.
