# Supplementary material for: First successful transcatheter valve-in-valve implantation into a failed mechanical prosthetic mitral valve after fracturing the discs: a case report
Source: Eur Heart J Case Rep. 2025 May 9;9(5):ytaf183. doi: 10.1093/ehjcr/ytaf183 (PMC12063098; doi:10.1093/ehjcr/ytaf183)
Supplement: ytaf183_Supplementary_Data [file ytaf183_supplementary_data.zip › Suppl. 2 Legend_R2.docx]

**Supplement 2**

1. **Summary Video_Valve-in-valve implantation after fracturing the discs**
2. **TAVI_5_ Ballon Fragment_B 29102020_110317 33.TIF**

Balloon fracturing the tilts, one fixed aside of the balloon

1. **TAVI_6_Sapien_kein Fragment_B 29102020_110436 34.TIF**

Sapien 3 before deployment in place, no tilt visible

1. **TAVI_7_Sapien_Impl_B 29102020_110526 37.JPG**

Sapien 3 deployment, balloon fully inflated

1. **TAVI_8_final_übersicht_B 29102020_111345 39.TIF**

Final chest X-ray
