# Supplementary material for: First successful transcatheter valve-in-valve implantation into a failed mechanical prosthetic mitral valve after fracturing the discs: a case report
Source: Eur Heart J Case Rep. 2025 May 9;9(5):ytaf183. doi: 10.1093/ehjcr/ytaf183 (PMC12063098; doi:10.1093/ehjcr/ytaf183)
Supplement: ytaf183_Supplementary_Data [file ytaf183_supplementary_data.zip › Supplementary material_1_Patient history.docx]

Supplementary material

**History and social background of the patient**

At the age of 4 years, a partial AV canal, an atrial septal defect and mitral cleft were corrected, and one year later the mitral valve was reconstructed. In 1996, the native mitral valve was replaced by a mechanical bileaflet valve (St. Jude 29mm). The postoperative course was complicated by severe bleeding and tamponade, which necessitated a re-thoracotomy. The operating report described a challenging anatomical access to the mitral valve due to fibrotic tissue, scar masses and distorted pathology. In 1993, she suffered an extensive embolic stroke of the left arteria cerebri medial and both arteriae cerebri posterior. As a consequence of this event she developed a severe drug-resistant multifocal epilepsy with psychomotorical and automotorical seizures as well as bilateral tonic-clonic seizures. Furthermore, she suffered from dissociative, psychogenic, non-epileptic seizures. In 2017, a vagus nerve stimulator (AspireSR Modell 106) was implanted, which reduced the number of epileptic seizures, but did also not achieve freedom of episodes.

The choice of therapeutic approach had to be made against the background of the complex personal, social and medical situation. She lives with her husband, who is also disabled, in an own apartment, supported by outpatient social and medical care. She is mobile with her rollator support and is protected by wearing a leather helmet at all times. Her legal and medical capacity is not restricted and she is fully capable of understanding, questioning and critically discussing medical issues. Nevertheless, her father (medical doctor and lawyer) and her aunt (lawyer) were
