# Supplementary figures and images for: First successful transcatheter valve-in-valve implantation into a failed mechanical prosthetic mitral valve after fracturing the discs: a case report
Source: Eur Heart J Case Rep. 2025 May 9;9(5):ytaf183. doi: 10.1093/ehjcr/ytaf183 (PMC12063098; doi:10.1093/ehjcr/ytaf183)

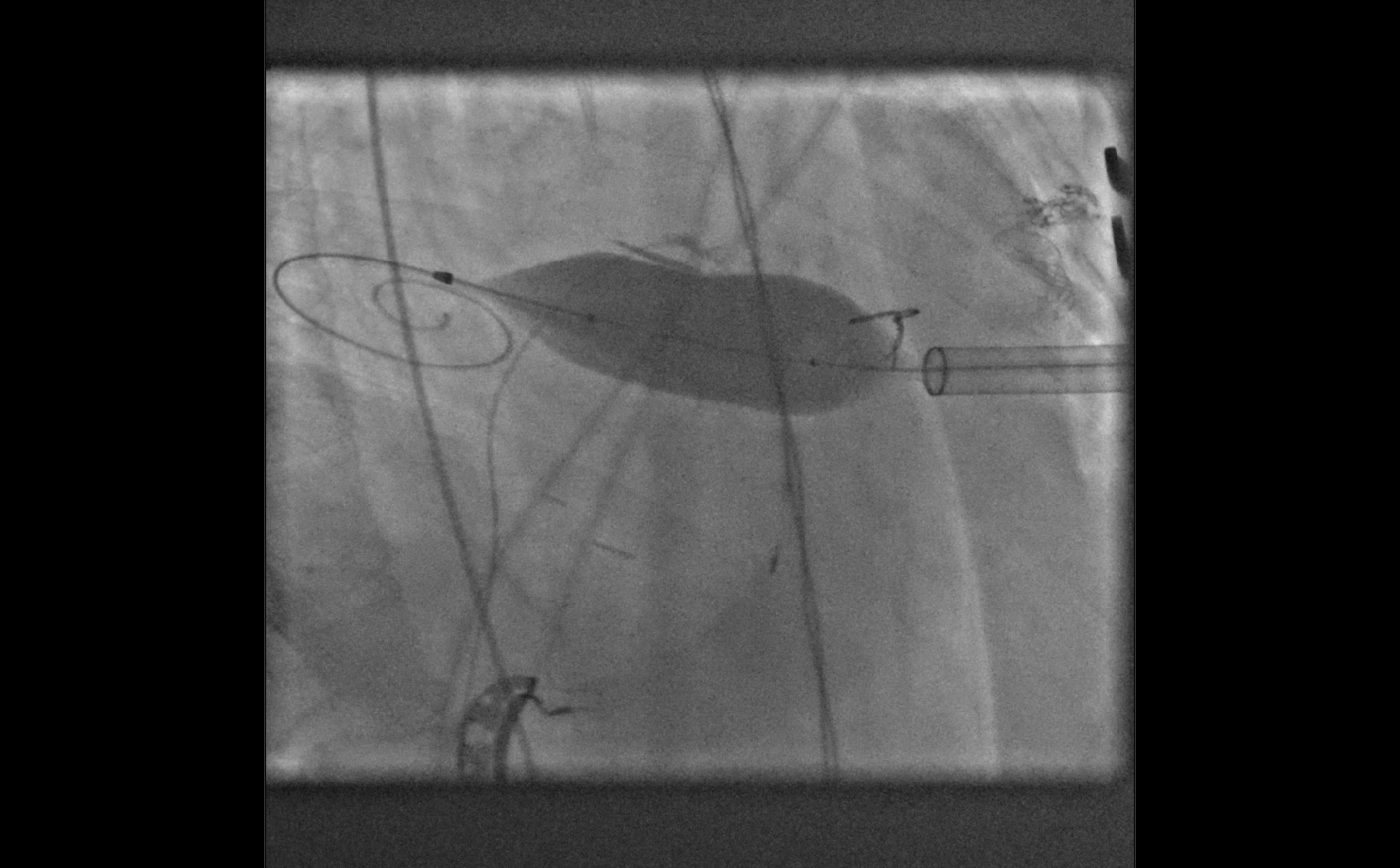

Supplement: ytaf183_Supplementary_Data [file ytaf183_supplementary_data.zip › Suppl _ Balloon fracuring the tilts.TIF]

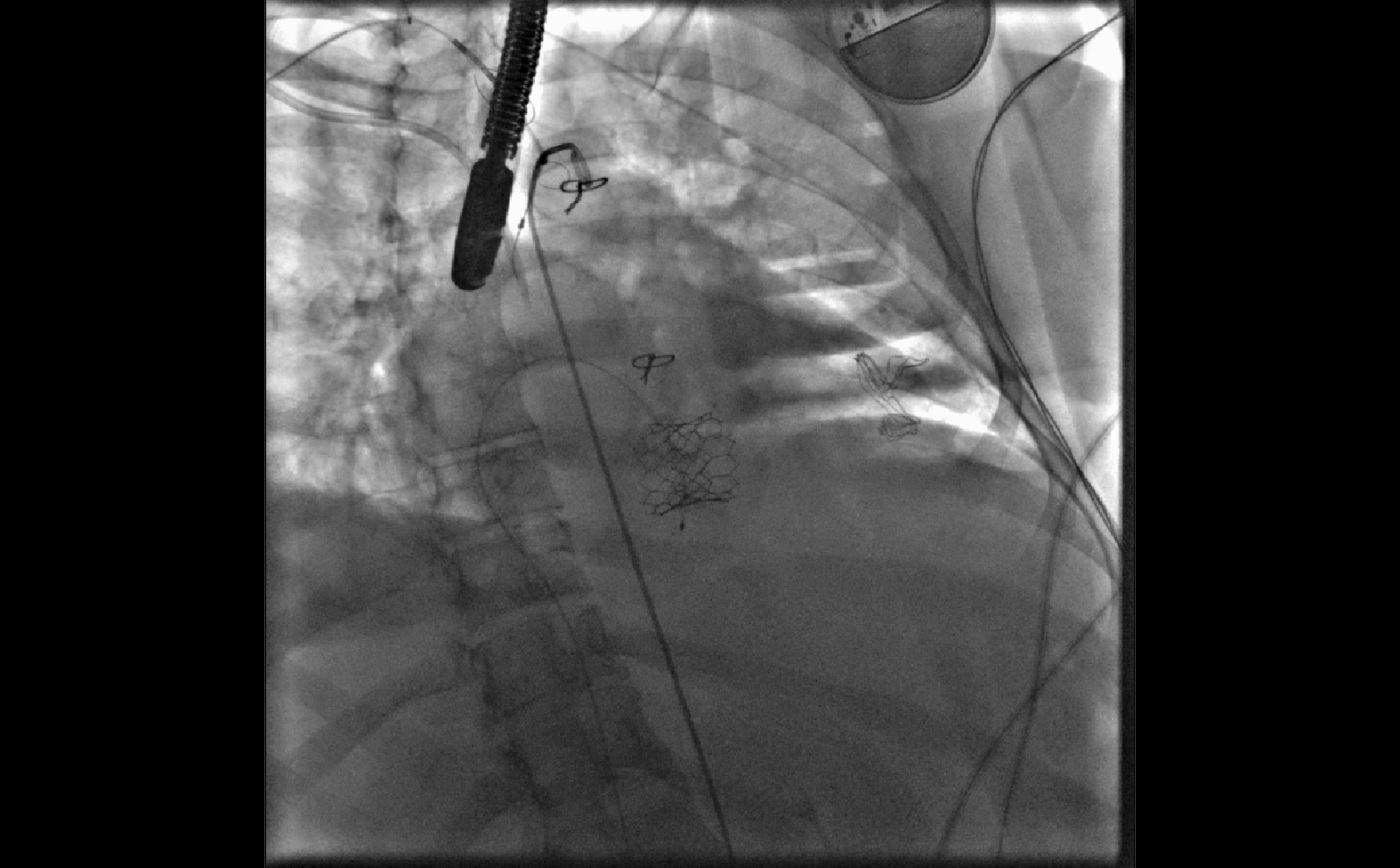

Supplement: ytaf183_Supplementary_Data [file ytaf183_supplementary_data.zip › Suppl _ Final chest Xray.TIF]

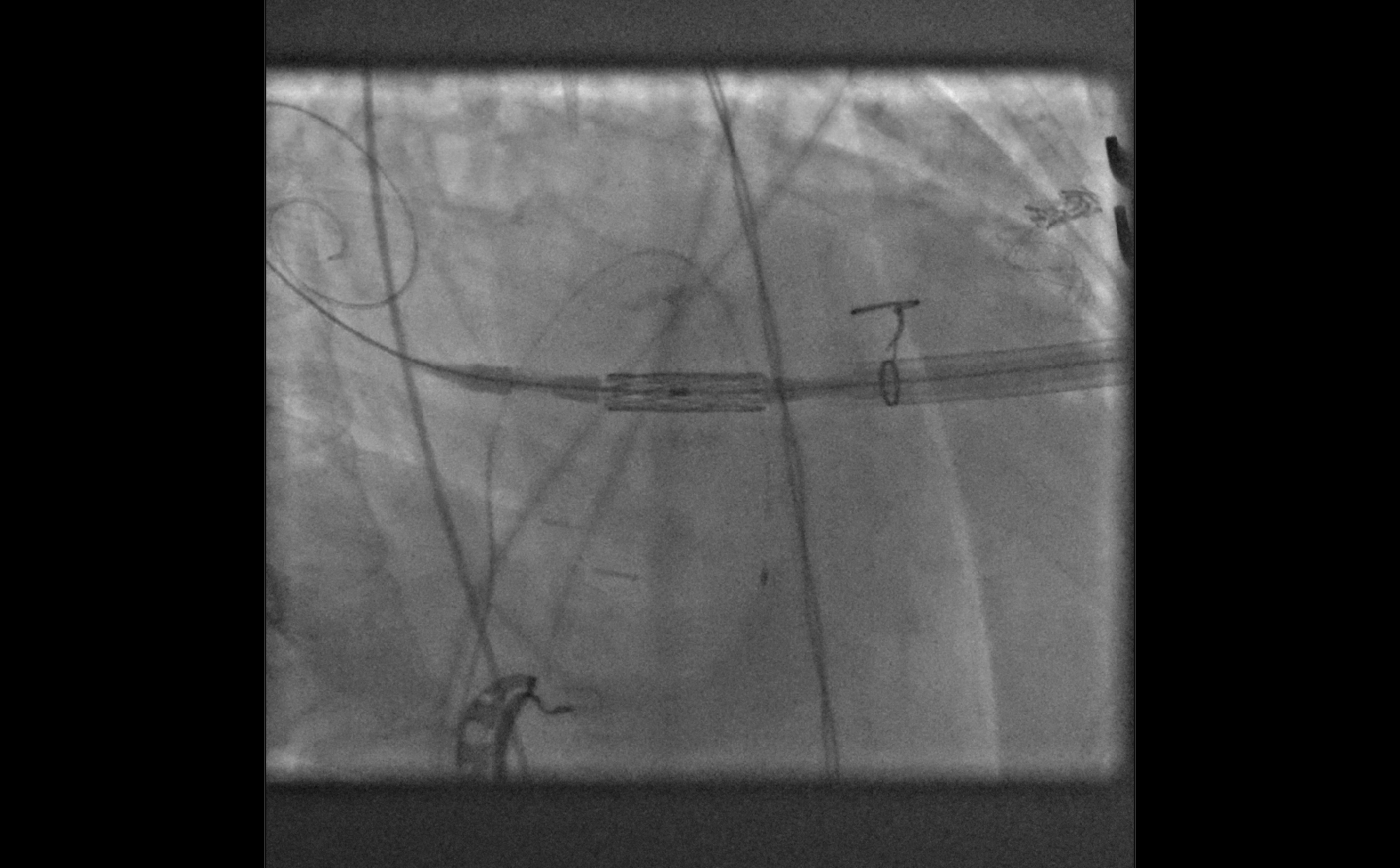

Supplement: ytaf183_Supplementary_Data [file ytaf183_supplementary_data.zip › Suppl _ Sapien 3 before deployment.TIF]

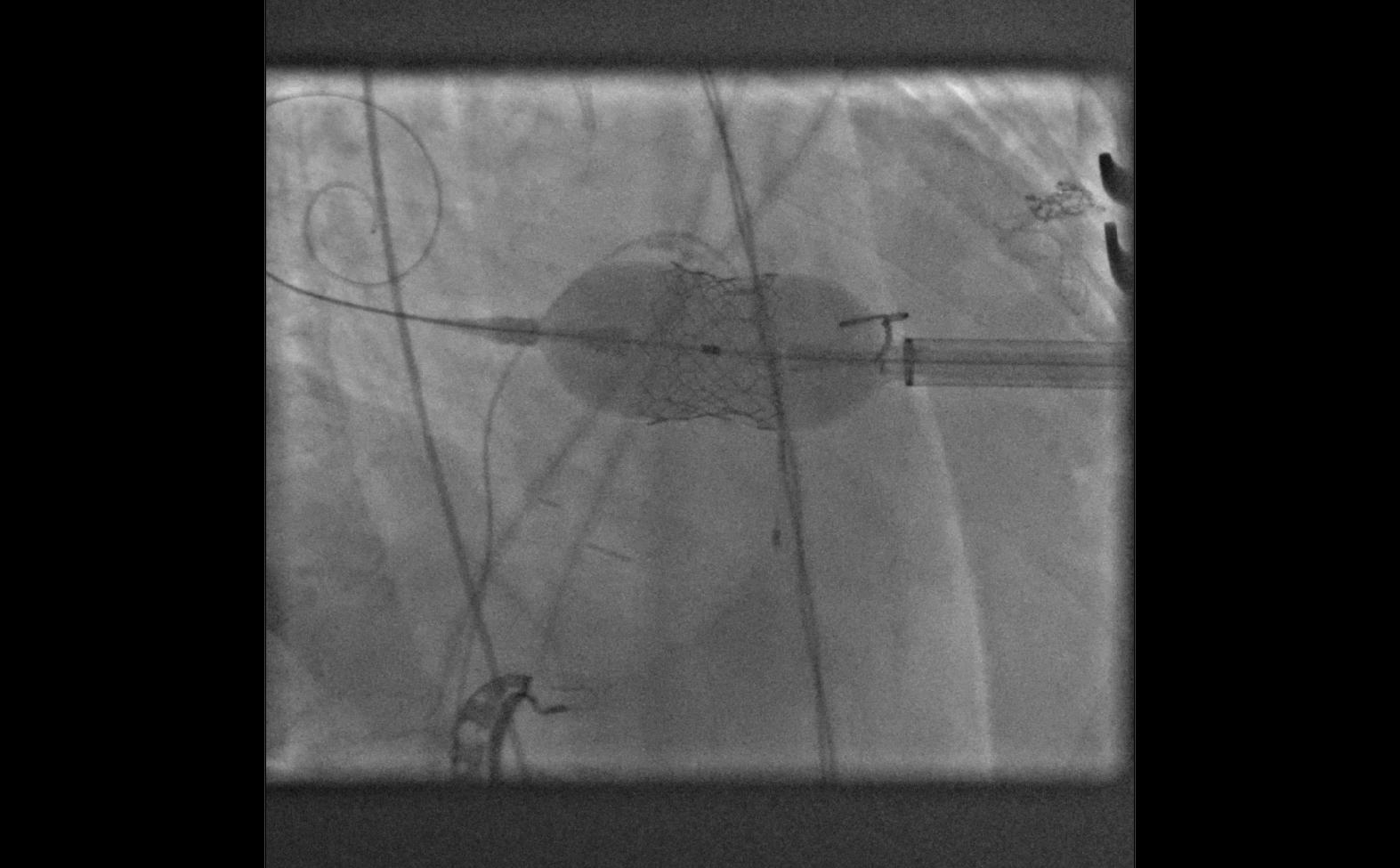

Supplement: ytaf183_Supplementary_Data [file ytaf183_supplementary_data.zip › Suppl _ Sapien 3 deployment and balloon fully inflated.JPG]
